# An integrative approach to predicting the functional effects of non-coding and coding sequence variation

**DOI:** 10.1093/bioinformatics/btv009

**Published:** 2015-01-11

**Authors:** Hashem A. Shihab, Mark F. Rogers, Julian Gough, Matthew Mort, David N. Cooper, Ian N. M. Day, Tom R. Gaunt, Colin Campbell

**Affiliations:** ^1^MRC Integrative Epidemiology Unit (IEU), University of Bristol, Bristol BS8 2BN, UK, ^2^Bristol Centre for Systems Biomedicine, University of Bristol, Bristol BS8 2BN, UK, ^3^Intelligent Systems Laboratory, University of Bristol, Bristol BS8 1UB, UK, ^4^Department of Computer Science, University of Bristol, Bristol BS8 1UB, UK and ^5^Institute of Medical Genetics, Cardiff University, Cardiff CF14 4XN, UK

## Abstract

**Motivation:** Technological advances have enabled the identification of an increasingly large spectrum of single nucleotide variants within the human genome, many of which may be associated with monogenic disease or complex traits. Here, we propose an integrative approach, named FATHMM-MKL, to predict the functional consequences of both coding and non-coding sequence variants. Our method utilizes various genomic annotations, which have recently become available, and learns to weight the significance of each component annotation source.

**Results:** We show that our method outperforms current state-of-the-art algorithms, CADD and GWAVA, when predicting the functional consequences of non-coding variants. In addition, FATHMM-MKL is comparable to the best of these algorithms when predicting the impact of coding variants. The method includes a confidence measure to rank order predictions.

Availability and implementation: The FATHMM-MKL webserver is available at: http://fathmm.biocompute.org.uk

**Contact:** H.Shihab@bristol.ac.uk** or **Mark.Rogers@bristol.ac.uk **or** C.Campbell@bristol.ac.uk

**Supplementary information:** Supplementary data are available at *Bioinformatics* online.

## 1 Introduction

Rapid technological advances and the falling costs of next-generation sequencing technologies have accelerated the identification of single nucleotide variants (SNVs) in the human genome (The 1000 Genomes Project, 2010). Ascertaining which of these are functional, against a background of neutral SNVs, promises to improve our understanding of the molecular mechanisms underpinning disease and complex traits. There is a plethora of computational algorithms capable of predicting whether SNVs are deleterious (Thusberg *et* *al.*, 2011). However, these algorithms are typically restricted to SNVs falling within protein-coding regions of the genome, with a particular focus on non-synonymous SNVs (nsSNVs). They are therefore incapable of assessing the consequences of a significant proportion of SNVs, given that the vast majority of SNV-trait associations fall within intergenic or intronic regions of the genome (Hindorff *et* *al.*, 2009). In this article, we propose a method for predicting the functional impact of both coding and non-coding SNVs.

Existing protein-based prediction algorithms tend to exploit evolutionary conservation when determining whether or not variants are functional. However, other potential sources for functional annotation are also now available from the Encyclopaedia of DNA Elements (ENCODE) consortium. Using several different technologies, ENCODE aims to identify all functional elements within the human genome, including transcribed non-coding RNAs, transcription factor binding sites and chromatin structure (The ENCODE Project Consortium, 2012). At the time of writing, ENCODE has produced approximately 1640 datasets describing the analysis of 24 different types of experiments in 147 cell lines, under various conditions (Qu and Fang, 2013). One of the major challenges faced by researchers is to successfully integrate the wealth of information contained within ENCODE to predict the functional consequences of SNVs.

Following our previous work (Shihab *et* *al.*, 2013b, a, 2014), we describe a machine learning approach (called FATHMM-MKL) that integrates functional annotations from ENCODE with nucleotide-based sequence conservation measures. When assessing the functional consequences of non-coding variants, we observe improved performance when compared with two recently proposed variant prediction algorithms: GWAVA (Ritchie *et* *al.*, 2014) and CADD (Kircher *et* *al.*, 2014), which as far as we are aware, are the only other proposed methods that can predict the functional consequences of non-coding variants. Furthermore, our method achieves comparable performance to CADD when predicting the functional impact of nsSNVs. A web-based implementation of FATHMM-MKL, including pre-computed predictions for the entire human genome and downloadable software, is available at http://fathmm.biocompute.org.uk.

## 2 Methods and materials

### 2.1 Datasets

We assembled two distinct datasets: our pathogenic dataset was constructed using heritable germ-line mutations from the Human Gene Mutation Database (Stenson *et* *al.*, 2014) (release 2013.4, subsequently denoted HGMD) and our control dataset was constructed using SNVs from the 1000 Genomes Project (The 1000 Genomes Project Consortium, 2012). This control dataset will contain many variants that are unannotated, and therefore it will likely contain some true positives. We will consider this issue in more detail in Section 3.3. Similarly, among positively labelled datapoints from the HGMD dataset, we could expect some true negatives: some neutral variants can be inherited alongside pathogenic variants due to genomic proximity, for example. Thus the positive class should be viewed as disease-associated variants that are enriched for functional impact as a set. The method we are to describe can be used with datasets for both inherited single nucleotide polymorphisms (SNPs) and somatic SNVs. The HGMD dataset is therefore an example of the former category.

For our analysis, we restricted our control dataset to SNVs having a global minor allele frequency ≥1% and further removed those that were also present in our pathogenic dataset. In order to assess the predictive utility of our method in both coding and non-coding regions of the genome, we further split our pathogenic and control datasets according to whether or not the variant introduces an amino acid substitution. We used 10 feature groups, denoted [*A*–*J*], which could be predictive of disease association and are therefore used to annotate out datasets using a customized pipeline. These feature groups are more fully described in the Supplementary material, but a short description is as follows:

- **46-Way Sequence Conservation**: based on multiple sequence alignment scores, at the nucleotide level, of 46 vertebrate genomes compared with the human genome.
- **Histone Modifications (ChIP-Seq)**: based on ChIP-Seq peak calls for histone modifications.
- **Transcription Factor Binding Sites (TFBS PeakSeq)**: based on PeakSeq peak calls for various transcription factors.
- **Open Chromatin (DNase-Seq)**: based on DNase-Seq peak calls.
- **100-Way Sequence Conservation**: based on multiple sequence alignment scores, at the nucleotide level, of 100 vertebrate genomes compared with the human genome.
- **GC Content**: based on a single measure for GC content calculated using a span of five nucleotide bases from the UCSC Genome Browser.
- **Open Chromatin (FAIRE)**: based on formaldehyde-assisted isolation of regulatory elements (FAIRE) peak calls.
- **Transcription Factor Binding Sites (TFBS SPP)**: based on SPP peak calls for various transcription factors.
- **Genome Segmentation**: based on genome-segmentation states using a consensus merge of segmentations produced by the ChromHMM and Segway software.
- **Footprints**: based on annotations describing DNA footprints across cell types from ENCODE.

### 2.2 Data integration

The resulting product of this data preparation is several large matrices comprising data from different feature groups, each of which can have different measurement scales. These feature groups can all indicate whether an SNV is functional or not, and hence we use a classifier based on multiple kernel learning (MKL). In MKL, different types of input data are encoded into kernel matrices, which quantify the similarity of data objects. A number of different methods have been proposed for deriving kernel matrices for different types of data objects, including data with discrete and continuous values, sequence data and graph data (Shawe-Taylor and Cristianini, 2004). With MKL, each constituent type of data is encoded into a corresponding base kernel $K_{\ell }$ (where ℓ=1,…,p if there are *p* feature groups), from which we can derive a composite kernel matrix $K=\sum_{\ell =1}^{p}\lambda_{\ell }K_{\ell }$ where $\sum_{\ell =1}^{p}\lambda_{\ell }=1$ and $\lambda_{\ell }\geq 0$. The $\lambda_{\ell }$ are *kernel weights*. This composite kernel can then be used with a kernel-based classifier, such as a support vector machine (SVM) (Campbell and Ying, 2011), which was the classifier used here. During the training phase, this approach requires determination of the learning parameters for the SVM in addition to the kernel weights. A variety of methods have been proposed for MKL (Gönen and Alpaydin, 2011), and this approach has been successfully demonstrated with various classification problems in bioinformatics, which use different types of input data (Ying *et* *al.*, 2009). By using all available data encoded into a set of kernels, MKL classifiers most frequently outperform a single kernel classifier constructed for one type of data. In addition, the kernel weights are adjusted according to the relative informative-ness of the different types of data: this enhances overall performance and interpretation of the model.

As further explained in the Supplementary Material, we also introduce a confidence measure associated with predicted class label. An SVM for binary classification has a decision function of the form sign(ϕ) with the sign of ϕ determining the predicted class. However, the magnitude |ϕ| is also a measure of the confidence in this class assignment. By fitting a sigmoid function, we convert ϕ into a confidence measure based on the posterior probability of a positive (pathogenic) outcome *P*(y = 1|ϕ) (Platt, 1999). Apart from indicating the reliability of a prediction, this confidence measure can be used to rank predictions and to further enhance predictive accuracy via cautious classification, i.e. by restricting predictions to high confidence instances, we achieve greater accuracy, but at the expense of only making predictions on a fraction of variants. Using a *P*-value (posterior probability value) cutoff, we can isolate a subset of predictions with higher reliability for subsequent experimentation. In order to compare predictive test accuracies, we report our results using receiver operating characteristic (ROC) curves and associated area under the curve (AUC) measures for both non-coding and coding variants.

### 2.3 Predicting the effects of non-coding variants

Our non-coding dataset was substantially imbalanced, yielding more than 6.7 million negative examples and 12 438 positive examples (annotated disease-causing mutations). As noted by Ritchie *et* *al.* (2014), examples from the HGMD database are not distributed uniformly across the genome, and it is unlikely that the genes associated with these positive examples represent an unbiased sample. To mitigate potential bias, and to facilitate a comparison with GWAVA, we used the strictest data selection procedure outlined in Ritchie *et* *al.* (2014), i.e. selecting negative examples that fall within a 1000-*nt* window of some positive example. This reduces our pool of negative examples from several million to 24 064. Furthermore, in order to assemble a training dataset for our MKL classifier, we required training examples with at least one value in each of our feature groups. Unfortunately, data can be absent from some feature groups for a given training example. Therefore, we could in principle use a larger number of feature groups, which would result in fewer training examples, or we could use a smaller number of feature groups, but with many more training examples. If we used a 10-feature group model [A–J], this requirement restricted our dataset to 1372 negative examples and just 913 positive examples. However, if instead we opted to use four feature groups (see the Supplementary data for how these groups were selected), this yielded a dataset comprising 5252 negative examples and 3063 positive examples for training and testing. As noted in the Supplementary data, the final version of our algorithm uses four feature groups [A–D].

We compared our method to two state-of-the-art methods for predicting non-coding deleterious mutations: GWAVA (Ritchie *et* *al.*, 2014) and CADD (Kircher *et* *al.*, 2014). CADD is a database of pre-computed scores. As with our method, CADD is based on use of a SVM classifier. CADD also integrates different annotations to create a single score (C-score), though it does not use an algorithmic procedure to weight different annotations according to relevance, unlike our approach. Though the C-scores derived within CADD quantify extent of pathogeneity, the derivation of these scores is distinct from the probability measures we introduce in this article. To ensure a fair comparison, we restricted our test data to those examples that appear in the CADD database. GWAVA is a random forest classifier that makes predictions for novel SNVs based on training data that are included with the software. Prediction on any of GWAVA’s training examples should be trivial and represents the training error, which does not estimate how well GWAVA generalizes to new variants. So we omitted those examples from our test set. This final filtering step reduced our test data from 8315 examples to 7623 examples (4591 negative and 3032 positive) for comparing our method with these alternatives. This resultant dataset provided enough examples to perform 5-fold cross-validation with 4800 examples for training and 1200 examples for testing in each fold.

In an experimental setting, we may also want to know the confidence associated with a particular prediction and the potential accuracy at a given confidence level. Therefore, we also associated a confidence measure with each prediction and considered restricting predictions to those instances where the associated confidence measure exceeds a given *P*-value cutoff, see the Supplementary data for details.

### 2.4 Predicting the effects of coding variants

In total, 87 518 coding examples were available, consisting of 67 305 positive and 20 213 negative examples. However, missing values in some feature groups and the requirement of a balanced dataset reduced the size of the training dataset. Using 10 feature groups, [A–J], it was possible to construct a balanced training dataset of 1073 positive and 1073 negative examples, from a set of 2714 positive and 1073 negative examples. If we use four feature groups [A–D], we could use a balanced set of 3000 positive and 3000 negative examples for training, from a set of 17 362 positive and 4853 negative examples. Using 5-fold cross-validation, the remaining data were used as the test set in both cases. We did not apply the restriction that negative examples must be located within a thousand nucleotides of positive examples (this would have resulted in too few negative training examples).

## 3 Results

### 3.1 Performance of the method: non-coding variants

**Fig. 1.**
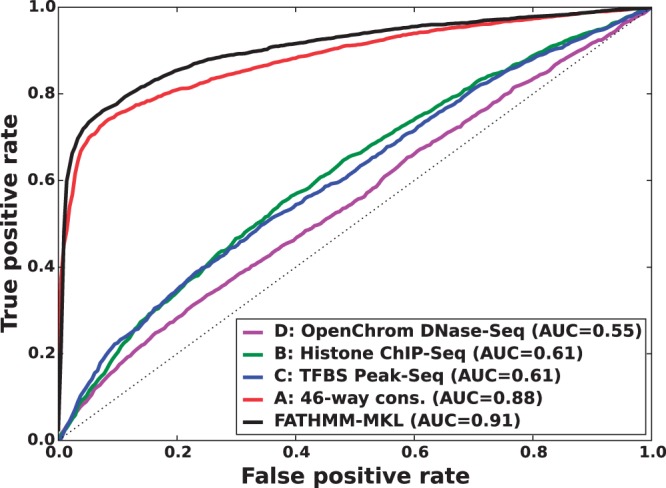
Five-fold cross-validation performance using the non-coding dataset and four feature groups (see Supplementary data [A–D]). ROC curves for FATHMM-MKL and classifiers using only one type of data. Even weak-performing feature group classifiers may contribute discriminatory information to the weighted aggregate MKL classifier, which outperforms any of the individual feature group classifiers

**Fig. 2.**
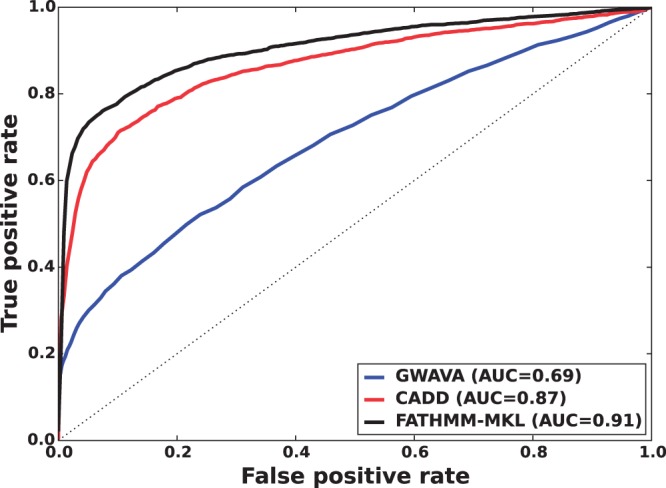
Five-fold cross-validation performance using the non-coding dataset and four feature groups (see Supplementary data [A–D]). ROC curves indicating that FATHMM-MKL yields substantially better predictive performance relative to CADD or GWAVA on the same examples

**Fig. 3.**
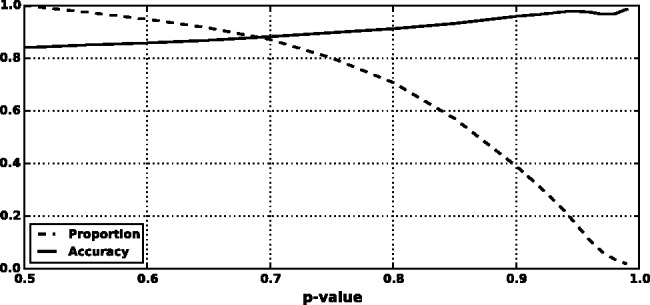
Five-fold cross-validation performance using the non-coding dataset and four feature groups (see Supplementary data [A–D]). Cautious classification yields nearly perfect performance as we increase the *P*-value cutoff (test accuracy as a fraction versus *P*-value cutoff)

**Fig. 4.**
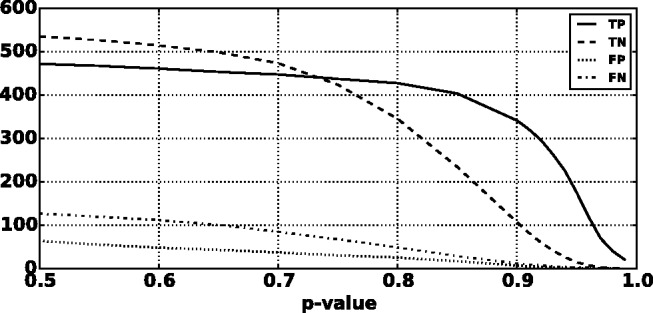
Five-fold cross-validation performance using the non-coding dataset and four feature groups (see Supplementary data [A–D]). The breakdown of true positive (TP), true negative (TN), false positive (FP) and false negative (FN) predictions for cautious classification shows that TP and TN predictions remain robust up to the highest *P*-value cutoffs (based on 5-fold cross-validation, the average number of test predictions from 1200 is 469.0 at a 90% cutoff and 195.4 at a 95% cutoff)

We found that a MKL model with the four most distinct feature groups (Figs. 1 and 2 with an AUC of 0.91) gave better performance than a model with all 10 feature groups (Supplementary Figs. S1 and S2 with an AUC for MKL of 0.85). We discuss the selection of these four feature groups in the Supplementary data. In experiments with this 4-feature group model, we found that feature group A, based on conservation scores from the alignment of 46 vertebrate genomes to the human genome, yielded the strongest performance of any individual kernel. It had an average AUC score of 0.88 (Fig. 1). The other feature groups yielded weaker performance with individual AUC scores ranging from 0.55 to 0.61. These scores are reflected in the MKL kernel weights (Supplementary Fig. S3). The weight for 46-way conservation is 0.71, whereas the second most informative feature group, TFBS Peak-Seq, receives a weight of 0.26. However, we found that MKL detects some additional discriminative power within the two weakest-performing feature groups with non-zero kernel weights of 0.004 and 0.03. The contribution of these weaker groups is evident from the overall MKL performance, which achieved an AUC score of 0.91 (Fig. 1). We obtained CADD scores by downloading files from their website and querying mutations using Tabix (Li, 2011); for GWAVA scores, we compared results from all three of the GWAVA models (*region*, *tss* and *unmatched*) and report those that yielded the highest accuracy in our tests. Our MKL classifier (FATHMM-MKL) yielded a significant performance improvement over both GWAVA (region) and CADD (Fig. 2). Performing a non-parametric test (simple binomial sign test) on the AUC scores across the 5-folds (FATHMM-MKL versus CADD), the difference in predictive accuracy is significant for all folds and the probability is 0.03125 that there is no statistical difference. When we apply cautious classification to our classifier, we can achieve near-perfect classification accuracy at the highest *P*-value cutoffs (Figs. 3 and 4). For predictions with 90% confidence or greater, the classifier achieves a 96% test accuracy while making predictions for nearly 40% of examples, while at 95% confidence, the accuracy increases to 98% with nearly 16% of examples having label predictions (Fig. 3).

### 3.2 Performance of the method: coding variants

**Fig. 5.**
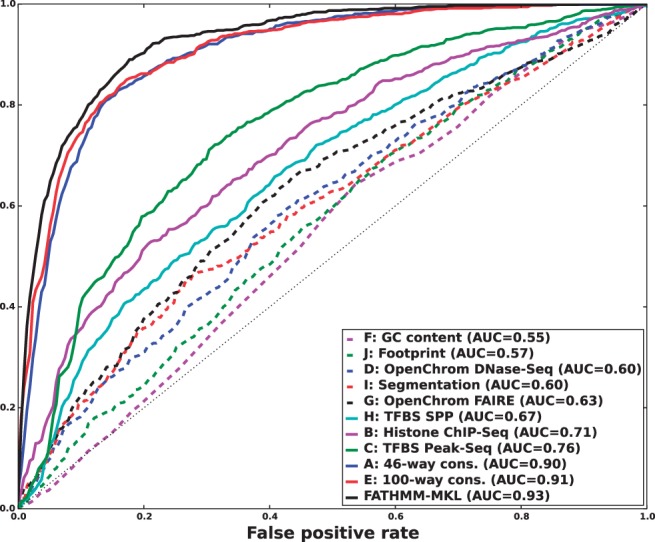
Five-fold cross-validation performance using the coding dataset and 10 feature groups (Supplementary [A–J]). ROC curves for FATHMM-MKL and for classifiers using only one type of data

**Fig. 6.**
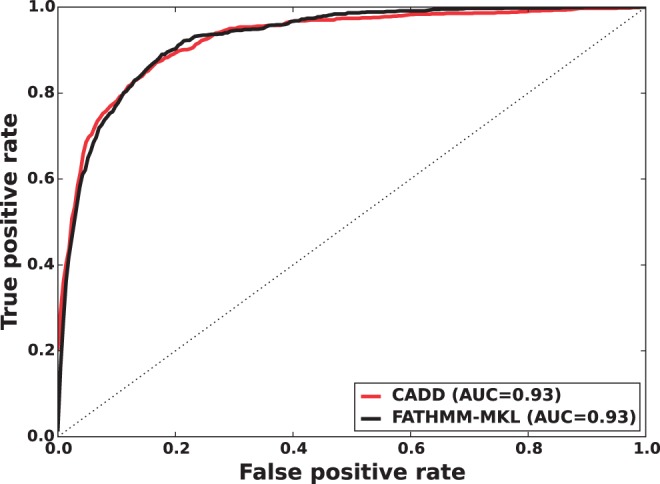
Five-fold cross-validation performance using the coding dataset and 10 feature groups (Supplementary [A–J]). ROC curves indicating that FATHMM-MKL yields comparable performance relative to CADD the same examples

Unlike our non-coding dataset, we found that an MKL model with all 10 discriminative feature groups (Figs. 5 and 6 with an AUC of 0.93) gave better performance than a model with the top four feature groups (Supplementary Figs. S4 and S5 with an AUC for MKL of 0.91). This superior performance with 10 feature groups was achieved despite using only 2146 training examples in contrast to the 6000 training examples used with the four-feature group model. We found that FATHMM-MKL was comparable to CADD on the same dataset (Fig. 6). Further, cautious classification gave similar curves to those for the non-coding data (Figs. 3 and 4), see Supplementary Figures S6 and S7.

### 3.3 Performance of method: prediction across all nucleotides in the human genome

#### 3.3.1 Prediction across the whole genome 

Next, we evaluated the prediction performance of FATHMM-MKL across a wider spectrum of the human genome (results for the entire human genome are available at the associated website). Across the wider genome, many ‘neutral’ (unannotated) variants may be true functional variants that are not reported because (i) they haven’t been tested against a relevant phenotype or (ii) their effects are subtle and haven’t been detected. In this section, we will explore the potential for FATHMM-MKL to correctly predict novel functional mutations and compare its behaviour with that of CADD and GWAVA across the entire human genome.

As noted earlier, our total of labelled variants consisted of 67 56 202 neutral and 12 438 positive non-coding examples, giving a total of 67 68 640. Of these, 65 07 401 had at least one of the four feature groups necessary to make a prediction (64 98 026 neutral and 9375 positive), which we used as our validation set. For many of these examples, we were not able to obtain data for all of our feature groups. In these cases, we used only the feature groups we had and rescaled the associated kernel weights by setting the missing kernel weights to 0 and the remaining weights ${\lambda \prime }_{\ell }=\lambda_{\ell }/\sum_{\ell }\lambda_{\ell }$. We compared our predictions for the same 6.5 million positions in the CADD database and to predictions from the GWAVA software tool. Both FATHMM-MKL and GWAVA yield scores in the range [0, 1], while the CADD database provides a raw decision function value along with an encoded ranking of its scores. In all cases, the higher the score, the greater the confidence of a functional mutation. Conversely, the lower the score, the greater the confidence that a mutation is neutral. Given this vastly unbalanced test dataset, we focused our evaluations on false-positive rates, using optimal thresholds for each method.

**Fig. 7.**
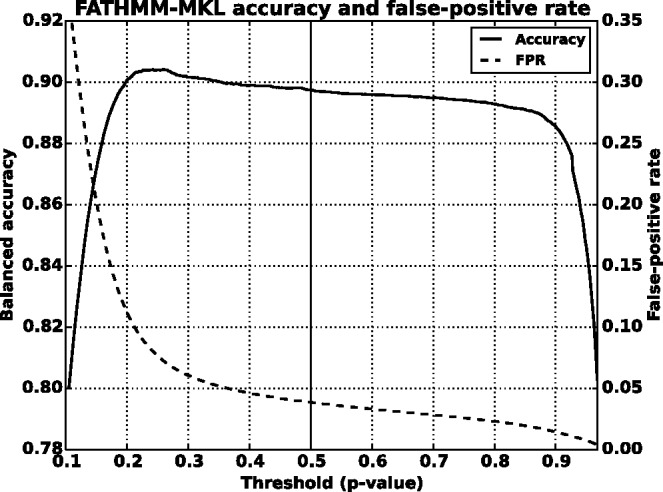
Evaluation of accuracy and false-positive counts for FATHMM-MKL on the full set of known examples from the 1000 Genomes and HGMD (evaluated on both coding and non-coding variants). FATHMM-MKL exhibits a nominal peak at 0.26, achieving balanced accuracy of 90.5% while yielding a false-positive rate of 7.2%. At its default threshold (0.5), FATHMM-MKL’s accuracy drops to 89.7% but with a dramatic drop in false-positive rate to 3.8%. Its accuracy remains over 88% up to a threshold of 0.92, with the false-positive rate dropping to just 1.2%

**Fig. 8.**
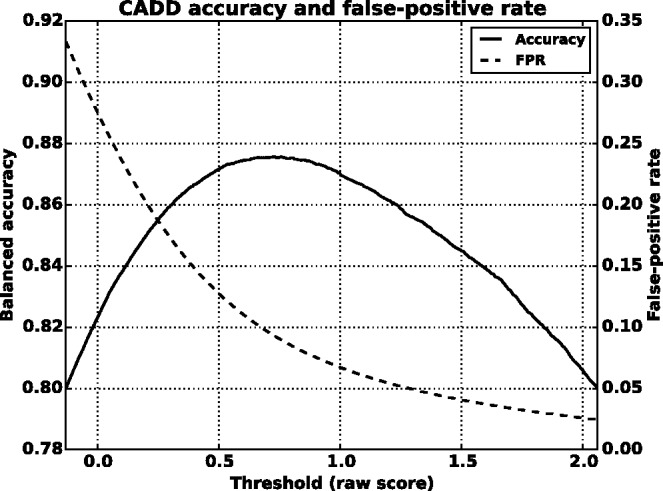
Evaluation of accuracy and false-positive counts for CADD, for comparison with FATHMM-MKL, depicted in Figure 7. CADD’s accuracy peaks at a threshold of 0.73, achieving balanced accuracy of 87.6% while yielding a false-positive rate of 9.3%. However, as its false-positive rate declines noticeably above this threshold, so does its accuracy

To find optimal thresholds, we iterated threshold values over the entire range of scores for each method. At each threshold we counted scores below the threshold as negative predictions and those at or above the threshold as positive. This allowed us to count true-positive, false-positive, true-negative and false-negative predictions for computing the balanced accuracy and the false-positive rate at each threshold. We also evaluated FATHMM-MKL at its intended threshold of 0.5. Figures 7 and 8 show the test accuracy curves for FATHMM-MKL and for CADD at different threshold values (raw scores for CADD and *P*-values for FATHMM-MKL). CADD’s accuracy peaks at a threshold of 0.73, achieving balanced accuracy of 87.6% while yielding a false-positive rate of 9.3%. FATHMM-MKL exhibits a nominal peak at 0.26, achieving balanced accuracy of 90.5% with a false-positive rate of 7.2%. At its default threshold of 0.5, FATHMM-MKL’s accuracy drops to 89.6% but with a dramatic drop in false-positive rate to 3.8%. GWAVA (region model) reached a peak accuracy of 67.7% at a threshold of 0.38, with a false-positive rate of 16.7% (Supplementary Fig. S8). We also compared the number of false-positive predictions at the highest confidence levels for each method. FATHMM-MKL yielded 4 84 780 false-positives at its optimum threshold, 2 58 840 false-positives at its default threshold of 0.5, 96 408 at *P*-values above 0.90, 50 357 above 0.95 and 1870 above 0.99. CADD had 6 29 370 false-positives at its optimal threshold, 3 54 264 false-positives ranked in the top 10% of its predictions, and 23 563 ranked in the top 1% (encoded rank values above 10 and 20, respectively).

**Fig. 9.**
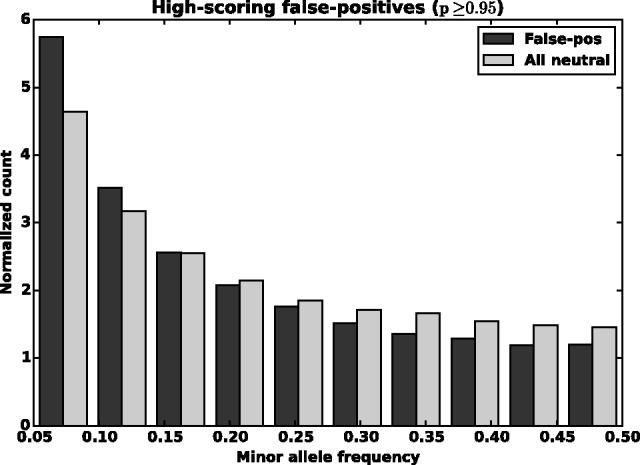
The normalized count versus minor allele frequency for false-positives (a labelled negative, predicted positive) and neutrals (a labelled negative, predicted negative)

We recall that for training we selected negative examples from regions within 1 K of some annotated deleterious example. Hence, the negative examples in our validation set consist almost entirely of examples outside these 1 K regions. In addition, the false-positive predictions on our validation set represent just 4–7% of negative examples. Thus, we consider the possibility that many of the false-positive predictions could be true positives, i.e. that they are mis-annotated. To explore this possibility further, in Figure 9, we plot the distribution of minor allele frequencies for our predicted high-scoring false-positives against the distribution of all neutral examples at a *P*-value confidence of 0.95 or higher. These high-scoring false-positives have a distribution that is shifted towards lower frequencies (this trend gradually diminishes as the *P*-value cutoff is reduced). Through evolution, common variants are unlikely to be associated with disease and it is rare variants, which are likely to be the main source of functional impact. An individual prediction should not be biased towards these rare variants. Consequently, the higher incidence of false positives among rarer variants suggests the false positive set in the data is actually enriched with some mislabelled true positives.

#### 3.3.2 Comparison with ClinVar data

**Fig. 10.**
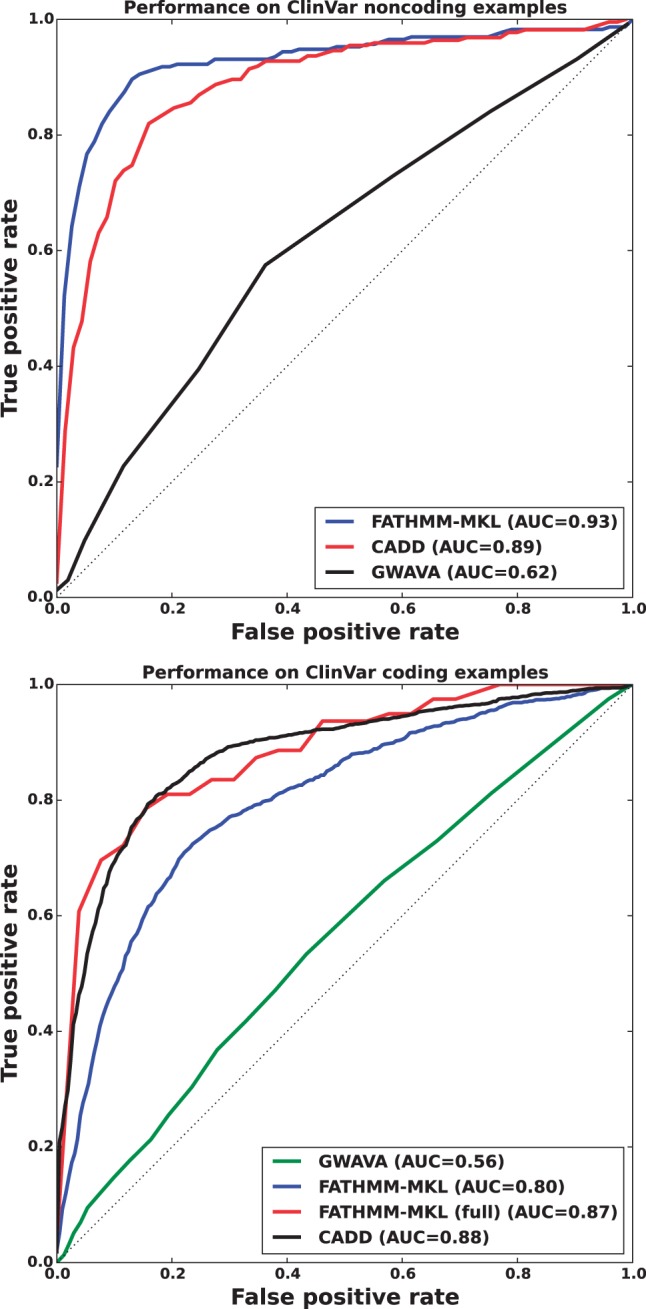
Performance of FATHMM-MKL, CADD and GWAVA on ClinVar examples for non-coding regions (top) and coding regions (bottom). As in our other tests, FATHMM-MKL performs substantially better than the other methods on non-coding examples, suggesting that it will generalize robustly to non-coding regions of the genome where the vast majority of novel deleterious SNVs are likely to reside. CADD’s coding classifier performed best overall, while both CADD and FATHMM-MKL outperformed GWAVA. When we considered only examples with data in all 10 feature groups, FATHMM-MKL’s performance was comparable to CADD’s (FATHMM-MKL full, bottom figure, red line)

We further evaluated FATHMM-MKL on data from the ClinVar database (Landrum *et* *al.*, 2013). This same resource was used to validate both GWAVA and CADD so was an ideal source of novel examples for comparing these methods (Ritchie *et* *al.*, 2014; Kircher *et* *al.*, 2014). Using the most recent dataset (August 8, 2014), we extracted records labeled either as benign or pathogenic (CLNSIG codes 2 and 5, respectively) and removed those found in our training data. This yielded sets of 647 non-coding and 3520 coding mutations that were unknown to FATHMM-MKL. Consistent with our other experiments, we found that FATHMM-MKL outperformed the other methods substantially on non-coding data (Fig. 10), with an AUC of 0.93 compared with 0.89 for CADD and 0.62 for GWAVA (region model). This gives us confidence that FATHMM-MKL will generalize robustly to other non-coding regions of the genome, where a vast number of novel deleterious SNVs are likely to be discovered. Classification on coding examples was somewhat weaker: FATHMM-MKL achieved a ROC of 0.80 compared with 0.88 for CADD and 0.56 for GWAVA (unmatched model). We speculated that this performance may be due to examples that were missing data for some of the FATHMM-MKL feature groups: of the 3520 coding examples, only 157 (4.5%) had data in all 10 feature groups. When we restricted our evaluation only to examples with data for all features, FATHMM-MKL’s performance improved substantially (red line, bottom of Fig. 10), once again comparable to CADD’s. This highlights a tradeoff between the accuracy we can achieve by using many sources of evidence, and a possible decrease in the number of confident predictions we make from those sources.

#### 3.3.3 Novel HGMD examples

Finally, the positive examples for our training dataset were derived from release 2013.4 of the Human Gene Mutation Database. During the interval after we constructed and tested the classifier, new datapoints were added to the HGMD (now release 2014.2), consisting of 2205 novel positive examples from coding regions and 401 from non-coding regions. This gives us an objective test of recall performance on unseen instances. Of these novel positive examples, 339 non-coding examples (85%) and 1955 coding examples (89%) are correctly predicted by our classifier at the default threshold. For some non-coding examples, we had no data in any of the feature groups and hence FATHMM-MKL could not make a prediction. When we consider only those examples where predictions can be made, these proportions increase to 88%. We also found that 34 of these novel positive examples were labeled negative in our training data, illustrating the potential prevalence of noise in the data. In summary, FATHHMM-MKL has distinct advantages over CADD and GWAVA and the above observations indicate that it provides reliable predictions with recall near 88%.

#### 3.3.4 FATHMM-MKL website

The associated website allows users to query our prediction database for novel deleterious mutations. In addition, we provide downloadable tables of ranked positive predictions (functional in disease) for three values of the *P*-value cutoff: at 0.95, 0.90 and at 0.26 (the former are subsets of the latter). Finally, we provide software for generating predictions given new data in at least one of our feature groups.

## 4 Discussion

The method we have outlined outperformed both GWAVA and CADD when predicting the functional consequences of non-coding sequence variants (Fig. 2 and Supplementary Fig. S2). In this case, better performance was achieved using a smaller number of feature groups ([A–D] in Fig. 1), as opposed to a broader range of feature groups ([A–J] in Supplementary Fig. S1). We also achieved good performance when predicting the functional impact of nsSNVs (Fig. 6). For coding sequence variants, better performance was achieved using *all* the feature groups ([A–J] in Fig. 5) instead of a smaller subset of feature groups (e.g. [A–D] in Supplementary Fig. S4). This out-performance was achieved despite the fact that the 10 feature group model used only 36% of the training examples available to the four feature group model. This suggests that a broad range of data sources are informative for classifying variants in coding regions. For predicting the functional impact of variants in non-coding regions, the opposite is suggested, with fewer types of data being truly informative. In both cases, the most informative indicator is whether or not the variant falls within regions which are highly conserved across species.

One advantage of our method is that it highlights the relative informative-ness of the different sources of data (e.g. Supplementary Fig. S3). Furthermore, the addition of a confidence measure also allows for the isolation of a smaller set of variants that have a higher confidence of correct functional impact assignment. This provides an intuitive way to rank predictions for subsequent analysis when discovering novel deleterious variants, as one may be able to survey only a small set of the most compelling variants. However, as noted in Section 3.3.2, incomplete data may restrict the feature groups we can use to make a prediction. Currently, we rescale kernel weights to accommodate these cases, but as our experiments with coding examples reveal, rescaling may not adequately compensate for missing features. We anticipate that rapidly growing data resources will mitigate this issue eventually, but immediate improvements in test accuracy are possible. For example, provided the number of feature groups remains small, we could learn sets of kernel weights specific to all possible combinations of feature groups. Feature groups had between 8 and 443 component features, with the strongest-performing group having the fewest features (46-way and 100-way conservation). It may be possible to improve performance using feature selection within these groups. In addition, rather than integrating component feature groups at the level of the data, via a composite kernel, it would be possible to integrate classifiers (each handling one feature group), via ensemble learning. Additional improvements are possible. For example, for non-coding regions, we could further exploit the sequence context of a variant to identify a possible functional element (e.g. a non-coding RNA site) and use this information to improve predictive accuracy.

In future projects, we shall investigate these potential improvements in addition to devising bespoke predictors for labelling variants in specific disease contexts, such as cancer. For prediction using coding variants, there are further sources of data that could be relevant to enhancing prediction performance. We will also experiment with these to establish if the proposed MKL method could be improved further, leading to out-performance over CADD.

## Supplementary Material

Supplementary Data
