# Supplementary material for: An integrative approach to predicting the functional effects of non-coding and coding sequence variation
Source: Bioinformatics. 2015 Jan 11;31(10):1536–43. doi: 10.1093/bioinformatics/btv009 (PMC4426838; doi:10.1093/bioinformatics/btv009)
Supplement: Supplementary Data [file supp_31_10_1536__index.html]

An Integrative Approach to Predicting the Functional Effects of Non-Coding and Coding Sequence Variation — An integrative approach to predicting the functional effects of non-coding and coding sequence variation — An integrative approach to predicting the functional effects of non-coding and coding sequence variation — Supplementary Data 

# An integrative approach to predicting the functional effects of non-coding and coding sequence variation

## Supplementary Data

files

**Files in this Data Supplement:**

- Supplementary Data - pdf file
