# Supplementary material for: An integrative approach to predicting the functional effects of non-coding and coding sequence variation
Source: Bioinformatics. 2015 Jan 11;31(10):1536–43. doi: 10.1093/bioinformatics/btv009 (PMC4426838; doi:10.1093/bioinformatics/btv009)
Supplement: Supplementary Data [file supp_btv009_supplementary.pdf]

BIOINFORMATICS

Vol. 00 no. 00 2014
Pages 1–5

Supplementary: An Integrative Approach to Predicting the Functional Effects of Non-Coding and Coding Sequence Variation

1 THE FEATURE GROUPS

We used the following feature groups to annotate each SNV in our pathogenic and control datasets:

A. **46-Way Sequence Conservation**: variants within highly conserved regions are likely to have more impact than those within high variability regions; therefore we used two measures of evolutionary conservation, namely PhastCons (Siepel *et al.*, 2005) and PhyloP (Pollard *et al.*, 2010) scores, obtained from the multiple sequence alignment (at the nucleotide level) of 46 vertebrate genomes to the human genome (Blanchette *et al.*, 2004). In addition to these scores, we constructed *ab initio* hidden Markov models (HMMs) representing these alignments using the HMMER software package (version 3.1b1) and extracted the relative probabilities of each nucleotide at the corresponding position within the alignment. Following our previous work (Shihab *et al.*, 2013b,a, 2014), we also included a measure of the magnitude of effect given the SNV (i.e. the log-odds ratio of observing both nucleotides).

B. **Histone Modifications (ChIP-Seq)**: we used ChIP-Seq peak calls for 14 histone modifications across 45 cell lines from ENCODE (The ENCODE Project Consortium, 2012).

C. **Transcription Factor Binding Sites (TFBS PeakSeq)**: we used PeakSeq (Rozowsky *et al.*, 2009) peak calls for 119 transcription factors across 77 cell lines from ENCODE.

D. **Open Chromatin (DNase-Seq)**: we used DNase-Seq peak calls across 119 cell lines from ENCODE.

E. **100-Way Sequence Conservation**: we used similar features to those described in A. but now obtained from the multiple sequence alignment of 100 vertebrate genomes to the human genome. We considered both 100-way (E) and 46-way sequence conservation (A) to highlight any gain which could be made by including more species in the comparison.

F. **GC Content**: we used a single measure for GC content calculated using a span of 5 nucleotide bases from the UCSC Genome Browser (Kent *et al.*, 2002).

G. **Open Chromatin (FAIRE)**: we used Formaldehyde-Assisted Isolation of Regulatory Elements (FAIRE) peak calls across 119 cell lines from ENCODE.

H. **Transcription Factor Binding Sites (TFBS SPP)**: we used SPP peak calls Kharchenko *et al.* (2008) for 119 transcription factors across 77 cell lines from ENCODE.

I. **Genome Segmentation**: we used 7 genome-segmentation states in 6 cell lines using a consensus merge of segmentations produced by the ChromHMM (Ernst and Kellis, 2010) and Segway software (Hoffman *et al.*, 2012).

J. **Footprints**: we used annotations describing DNA footprints across 41 cell types from ENCODE.

The number of features within each of these feature groups, and the type of feature used, is presented in Table 1

Group	Description	# Features	Type
A	46-way conservation	8	continuous
B	Histone ChIP-Seq	190	continuous
C	TFBS PeakSeq	443	continuous
D	Open chromatin DNase-Seq	122	discrete
E	100-way conservation	8	continuous
F	GC content	1	discrete
G	Open chromatin FAIRE	19	continuous
H	TFBS SPP	443	continuous
I	Genome segmentation	6	categorical
J	Footprints	41	continuous

Table 1. The features represented by each feature group, in terms of number of features and data type. Our ten feature groups have up to 443 features that may be continuous, discrete or categorical. Continuous and discrete features were encoded directly into linear kernels. Group I (Genome segmentation) has only categorical features, with 7 categories that we convert to 7-bit binary representations (42 features in total) for our kernel matrix representation of the data.

2 THE METHOD

We used a kernel-based classifier (Shawe-Taylor and Cristianini, 2004; Schölkopf and Smola, 2002), encoding each feature group into an appropriate base kernel. To avoid estimation of a kernel parameter we used linear kernels (Shawe-Taylor and Cristianini, 2004; Schölkopf and Smola, 2002), with use of a soft margin (see below) to cover the possibility of non-separable data. Continuous and integer-valued features were encoded directly into these linear kernels. Group **I** (Genome segmentation) had the only categorical features, with 7 different categories in all. These 7 categories were converted to 7-bit binary representation (hence $6 \times 7 = 42$ features in all) which was encoded via a linear kernel. Kernel matrices can be constructed for a very wide class of data objects, beyond the types of data described here. This includes sequence, tree-structured data and graph data (Shawe-Taylor and Cristianini, 2004)

Thus each constituent type of data was encoded into a corresponding base kernel $\mathbf{K}_\ell$, from which we derived the composite kernel matrix $\mathbf{K}_c = \sum_{\ell=1}^{p} \lambda_\ell \mathbf{K}_\ell$ where $\sum_{\ell=1}^{p} \lambda_\ell = 1$

© Oxford University Press 2014.

and $\lambda_\ell \geq 0$ (where $\ell = 1, \ldots, p$ ranges over the p types of data). For example, for the non-coding dataset with four feature groups (groups [A-D]), given in the paper, the spectrum of kernel weights, λ_ℓ, is depicted in Supporting Fig. 3.

The learning parameters in the classifier, α_i (where $i = 1, \ldots, m$ ranges over the m training examples in the data) and the kernel weights, λ_ℓ were found using SimpleMKL (Rakotomamonjy *et al.*, 2008). Having found the parameters and the composite kernel via the kernel weights, the predicted label of a new input $\mathbf{z}$ is given by the sign of $\phi(\mathbf{z}) = \sum_{j=1}^{m} \alpha_j y_j \mathbf{K}_c(\mathbf{x}_j, \mathbf{z}) + b$ where the offset b is found from:

$$b = -\frac{1}{2}\left[\max_{\{i|y_i=-1\}}\left(\sum_{j=1}^{m}\alpha_j y_j \mathbf{K}_c(\mathbf{x}_j,\mathbf{x}_i)\right) + \min_{\{i|y_i=+1\}}\left(\sum_{j=1}^{m}\alpha_j y_j \mathbf{K}_c(\mathbf{x}_j,\mathbf{x}_i)\right)\right]$$

During the training process, to determine the results presented in Fig. 1 and Fig. 2, we split the training data into 5 folds to identify an optimal C-value (a L1-soft margin parameter, see Campbell and Ying (2011) for details) and the kernel weights for the composite kernel. In each fold, we used SimpleMKL (Rakotomamonjy *et al.*, 2008) to find the optimal kernel weights, using C-values in the range $[10^{-4}, 10^{4}]$ as well as using a hard-margin (Campbell and Ying, 2011). In some cases, we also added a small ridge constant (10^{-8}) along a kernel matrix diagonal to ensure convergence during training. For testing, we then used the C-value and weights that yielded the highest accuracy on the held-out component of the training data. From this process, we selected $C = 0.01$ and the kernel weights by averaging across the folds in the training data, and used these results for evaluation of performance on the held-out test data.

With Fig. 3 and Fig. 4, we further used a confidence measure, associated with the class assignment. The above described MKL method has an intrinsic confidence measure, namely, $\phi(\mathbf{z})$. The larger the absolute value of $\phi(\mathbf{z})$ the greater the degree of confidence in the predicted label. We could relate $\phi(\mathbf{z})$ to a probability measure by fitting a sigmoidal probability function (Platt, 1999). Thus, for binary classification, we fit the sigmoid $p(y = +1|\phi) = [1 + \exp(A\phi + b)]^{-1}$. With class labels $y_i \in \{+1, -1\}$ and letting $t_i = 0.5(y_i + 1)$ the parameters A and B could be found by minimizing the negative log likelihood of the training data via the cross entropy error function (Platt, 1999):

$$\min_{A,B}\left[-\sum_i t_i \log(p_i) + (1 - t_i)\log(1 - p_i)\right]$$

where p_i is the sigmoid probability function evaluated from $\phi(\mathbf{x}_i)$. We used the Levenberg-Marquardt algorithm (Nocedal and Wright, 2000) to perform this minimization.

3 MODEL INTERPRETATION

3.1 Non-Coding Variants

In Supporting Fig. 1, we present the ROC curves and AUC scores for classifiers based on each component feature group and FATHMM-MKL (using all 10 feature groups $[A - J]$). As seen in Supporting Fig. 2, FATHMM-MKL outperforms GWAVA (Ritchie *et al.*, 2014) and CADD (Kircher *et al.*, 2014). However, performance is poorer than results for the four-feature-group model (Fig. 1). Note that the cross-validation data for the ten-group classifier is a subset of the data for the four-group classifier, so results are slightly different. To choose the 4 feature groups for the latter model, we used the AUC scores for the single feature group classifiers in Supporting Fig. 1.

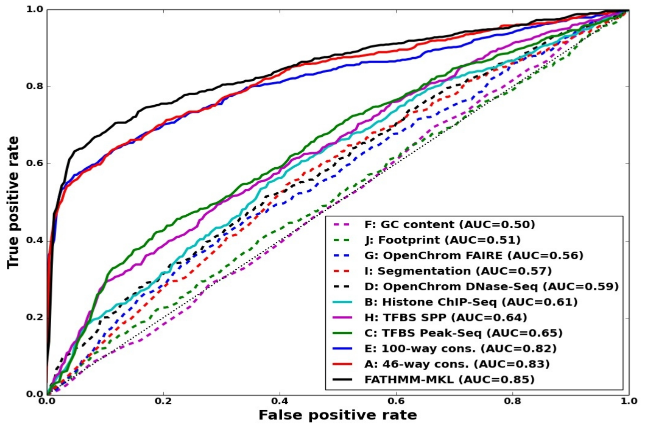

Supporting Fig. 1. Five-fold cross-validation performance for classifiers using a single source of data and FATHMM-MKL using the **non-coding** dataset. ROC curves for FATHMM-MKL and for classifiers using only one type of data.

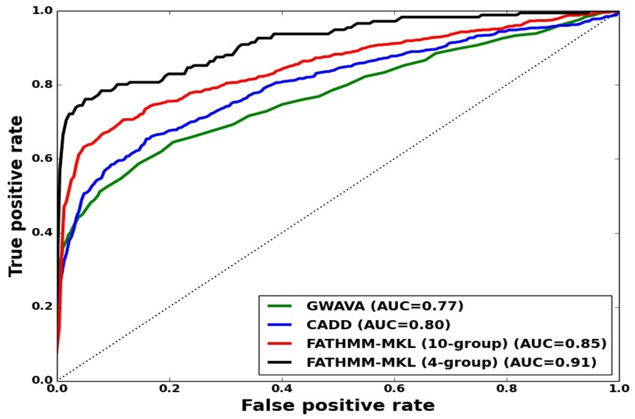

Supporting Fig. 2. Five-fold cross-validation performance for classifiers using a single source of data and FATHMM-MKL using the **non-coding** dataset and all ten feature groups. These ROC curves indicate that the two FATHMM-MKL classifiers yield better predictive performance relative to CADD or GWAVA, for the same set of training and test examples. Further, it is evident that the four-group FATHMM-MKL classifier substantially outperforms the ten-group classifier on non-coding examples.

For the non-coding dataset with 4 feature groups, the spectrum of kernel weights is depicted in Supporting Fig. 3.

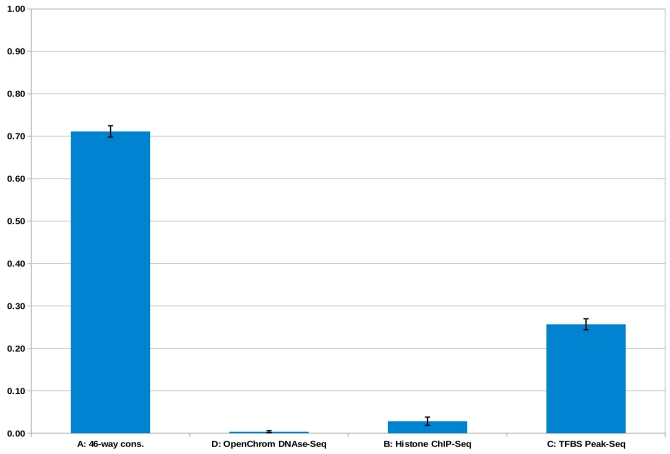

Supporting Fig. 3. The spectrum of kernel weights, λ_ℓ using a 4 feature group model on our **non-coding** dataset. Here, the height of each bar indicates the relative weighting given to that source of data by the MKL method.

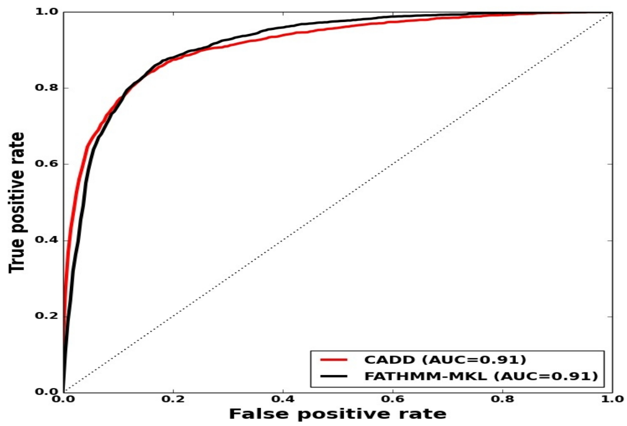

Supporting Fig. 5. Five-fold cross-validation performance for FATHMM-MKL and other classifiers, using the **coding** dataset, with the 4 feature groups [A-D]. Comparison of FATHMM-MKL and CADD on the same dataset.

3.2 The coding dataset

For the coding dataset, and using 4 feature groups $[A - D]$, we get an AUC score of 0.91, whereas with $[A - H]$ we get an AUC score of 0.93, despite using $2,146$ training examples as against $6,000$ training examples when using only [A-D] as the feature groups. In Supporting Fig. 4, we give the ROC curves and AUC scores for the 4 feature group dataset. Performance with this model is similar to that for CADD (Supporting Fig. 5) when evaluated on the same training and test sets.

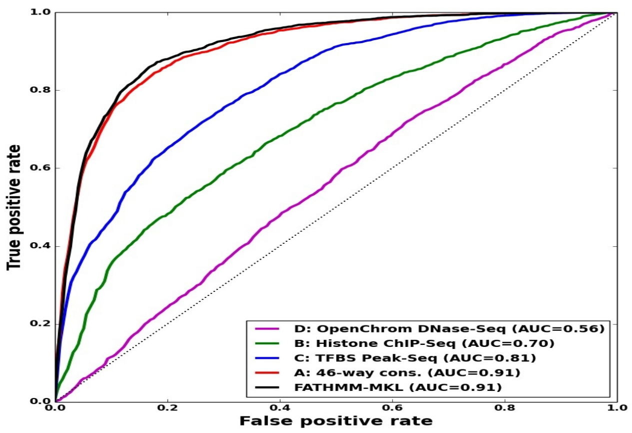

Supporting Fig. 4. Five-fold cross-validation performance for FATHMM-MKL and other classifiers, using the **coding** dataset, with the 4 feature groups [A-D]. ROC curves for FATHMM-MKL and for classifiers using only one type of data.

3.3 Conservation features

The feature groups based on 100-way and 46-way multiple sequence alignments (MSAs) provided by far the best performance for both noncoding and coding predictions (Figures 1 and 5, Supporting Figures 1, 3 and 4). To assess the features in these groups, we looked at the individual feature weights for SVMs trained on these groups. Each of these groups uses the same set of features:

- PhastCons score: a score based on multiple sequence alignments (MSAs) and a phylogenetic tree. It is similar to FATHMM but uses a phylogenetic HMM to account for distances between species and it accounts for conservation in the region surrounding each position.
- PhyloP score: as with PhastCons this score uses a phylogenetic HMM to assess evolutionary distances, but does not account for regional conservation.
- MSA depth: number of species used in the FATHMM MSA. The more species included in an alignment, the greater our confidence in the resulting conservation score.
- P_w: FATHMM emission probability for the wild-type nucleotide
- P_m: FATHMM emission probability for the mutant nucleotide
- Difference, Absolute diff.: the difference (absolute difference) between P_w and P_m. The greater the difference, the greater a mutation's potential impact.
- Ratio: the unweighted FATHMM score, which is the logs-odds ratio of P_w and P_m.

For noncoding predictions we find that the PhastCons score based on phylogenetic HMMs yields the highest single weight (1.17), while three of the FATHMM components (MSA depth, P_w, Absolute diff.) and the PhyloP score also contribute substantially

Feature	Noncoding 100-way	Coding 100-way	Coding 46-way
PhastCons	1.1671	0.5384	0.5815
PhyloP	0.2750	0.3471	0.3900
MSA depth	0.1958	0.2641	0.4254
P_w	-0.1114	-0.0566	-0.0497
P_m	-0.0494	-0.3254	-0.2677
Difference	0.0220	-0.0498	-0.0432
Absolute diff.	-0.1287	-0.0778	-0.0350
Ratio	-0.0456	-0.0303	-0.0828

Table 2. Feature weights for 100-way and 46-way conservation feature groups that provide the best discrimination in noncoding and coding predictions. SVM models trained on the same data used for the full MKL kernels yield the feature weights shown above. In sum the FATHMM components contribute substantially to noncoding predictions, but the PhastCons scores were the single most informative feature. In coding regions the weights are more evenly distributed between the three main methods.

to the group's performance. In coding predictions the weights are distributed more evenly across the three methods.

4 CAUTIOUS CLASSIFICATION WITH THE CODING DATA SET

For the non-coding dataset, we present results for the test accuracy versus a cutoff p-value for the confidence measure (see above), in Fig. 3 and Fig. 4. We pursued a similar study for the coding dataset and using the [A-D] datasets. The result is given in Supporting Fig. 6 and Supporting Fig. 7

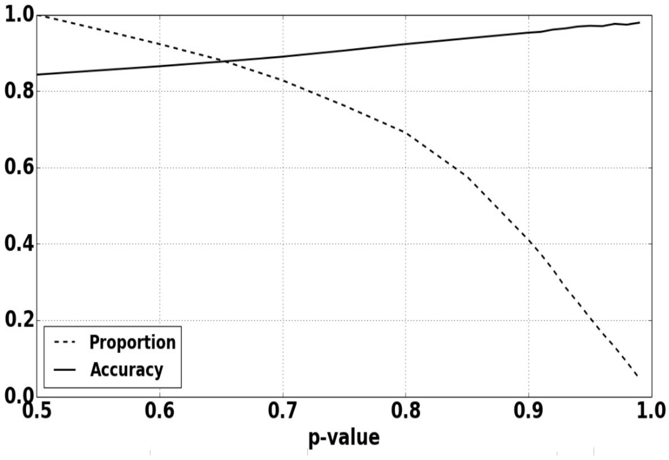

Supporting Fig. 6. Cautious classification with the **coding** dataset. Solid curve: test accuracy (y-axis) as a fraction versus p-value cutoff (x-axis). Dashed curve: proportion of predictions made as a fraction of the test set.

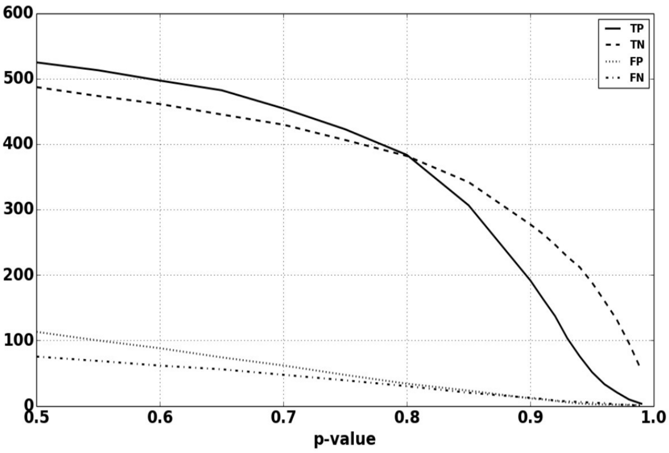

Supporting Fig. 7. Cautious classification with the **coding** dataset. The breakdown of true positive (TP), true negative (TN), false positive (FP) and false negative (FN) predictions.

5 FALSE-POSITIVE ANALYSIS ON GWAVA

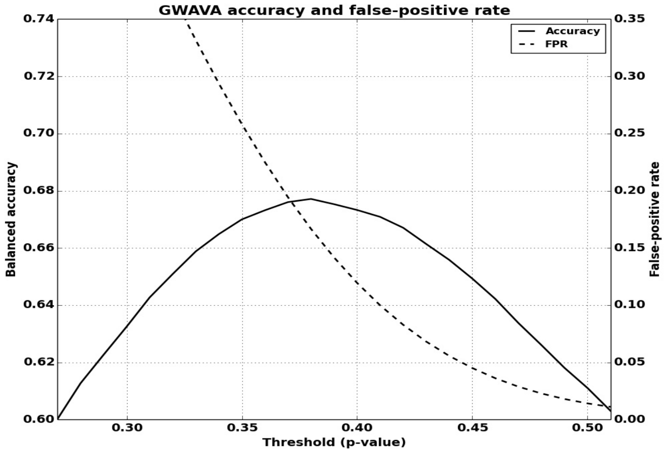

Supporting Fig. 8. Evaluation of accuracy and false-positive counts for GWAVA, for comparison with CADD and FATHMM-MKL, depicted in Figure 7. In this experiment, GWAVA's accuracy and false-positive rate are not competitive with the other two methods: its accuracy peaks at a threshold of 0.38, with balanced accuracy of 67.7% and a false-positive rate of 16.7%.

REFERENCES

Blanchette, M., Kent, W., Riemer, C., Elnitski, L., Smit, A., Roskin, K., Baertsch, R., Rosenbloom, K., Clawson, H., Green, E., Haussler, D., and Miller, W. (2004). Aligning multiple genomic sequences with the threaded blockset aligner. *Genome Research*, **14**, 708–715.

Campbell, C. and Ying, Y. (2011). *Learning with Support Vector Machines*. Morgan and Claypool.

Ernst, J. and Kellis, M. (2010). Discovery and characterization of chromatin states for systematic annotation of the human genome. *Nature Biotechnol.*, **28**, 817–825.

Hoffman, M. M., Buske, O., Wang, J., Weng, Z., Bilmes, J., and Noble, W. (2012). Unsupervised pattern discovery in human chromatin structure through genomic segmentation. *Nature Methods*, **9**, 473–476.

Kent, W. J., Sugnet, C., Furey, T., Roskin, K., Pringle, T., Zahler, A., and Haussler, D. (2002). The human genome browser at ucsc. *Genome Research*, **12**, 996–1006.

Kharchenko, P. V., Tolstorukov, M. Y., and Park, P. J. (2008). Design and analysis of chip-seq experiments for DNA-binding proteins. *Nature Biotechnology*, **26**, 1351–1359.

Kircher, M., Witten, D., Jain, P., O'Roak, B., Cooper, G., and Shendure, J. (2014). A general framework for estimating the relative pathogenicity of human genetic variants. *Nature Genetics*, **46**, 310–315.

Nocedal, J. and Wright, S. (2000). *Numerical Optimization*. Springer.

Platt, J. (1999). *Advances in Large Margin Classifiers*, chapter Probabilistic Outputs for Support Vector Machines and Comparisons to Regularized Likelihood Methods, pages 61–74. MIT Press.

Pollard, K. S., Hubisz, M., Rosenbloom, K., and Siepel, A. (2010). Detection of non-neutral substitution rates on mammalian phylogenies. *Genome Research*, **20**, 110–121.

Rakotomamonjy, A., Bach, F., Canu, S., and Grandvalet, Y. (2008). Simplemkl. *Journal of Machine Learning Research*, **9**, 2491–2521.

Ritchie, G. R. S., Dunham, I., Zeggini, E., and Flicek, P. (2014). Functional annotation of noncoding sequence variants. *Nature Methods*, **11**, 294–296.

Rozowsky, J., Euskirchen, G., Auerbach, R., Zhang, Z., Gibson, T., Bjornson, R., Carriero, N., Snyder, M., and Gerstein, M. (2009). Peakseq enables systematic scoring of chip-seq experiments relative to controls. *Nature Biotechnology*, **27**, 66–75.

Schölkopf, B. and Smola, A. J. (2002). *Learning with kernels: Support Vector Machines, regularization, optimization, and beyond*. MIT Press.

Shawe-Taylor, J. and Cristianini, N. (2004). *Kernel Methods for Pattern Analysis*. Cambridge University Press.

Shihab, H. A., Gough, J., Cooper, D. N., Day, I. N., and Gaunt, T. R. (2013a). Predicting the functional consequences of cancer-associated amino acid substitutions. *Bioinformatics*, page btt182.

Shihab, H. A., Gough, J., Cooper, D., Stenson, P., Barker, G., Edwards, K., Day, I., and Gaunt, T. (2013b). Predicting the functional, molecular, and phenotypic consequences of amino acid substitutions using hidden markov models. *Human Mutation*, **34**, 57–65.

Shihab, H. A., Gough, J., Mort, M., Cooper, D. N., Day, I. N., and Gaunt, T. R. (2014). Ranking non-synonymous single nucleotide polymorphisms based on disease concepts. *Human Genomics*, **8**(1), 11.

Siepel, A., Bejerano, G., Pedersen, J., Hinrichs, A., Hou, M., Rosenbloom, K., Clawson, H., Spieth, J., Hillier, L., Richards, S., Weinstock, G., Wilson, R., Gibbs, R., Kent, W., Miller, W., and Haussler, D. (2005). Evolutionarily conserved elements in vertebrate, insect, worm, and yeast genomes. *Genome Research*, **15**, 1034–1050.

The ENCODE Project Consortium (2012). An integrated encyclopedia of DNA elements in the human genome. *Nature*, **489**, 57–74.
